# Sex differences in the dynamics of the distance between the talus and lateral malleolus during the stance phase of gait

**DOI:** 10.1007/s10396-025-01577-6

**Published:** 2025-09-18

**Authors:** Noriaki Maeda, Satoshi Onoue, Yasunari Ikuta, Satoshi Arima, Honoka Ishihara, Ayano Ishida, Andreas Brand, Yuichi Nishikawa, Makoto Asaeda, Tomoyuki Nakasa, Nobuo Adachi, Tsubasa Tashiro

**Affiliations:** 1 Department of Sports Rehabilitation, Graduate School of Biomedical and Health Sciences Division of Integrated Health Sciences, Hiroshima University, 1-2-3 Kasumi, Minami-ku, Hiroshima, 734-8553 Japan; 2 Department of Orthopaedic Surgery, Graduate School of Biomedical and Health Sciences, Hiroshima University, Hiroshima, Japan; 3 Sports Medical Center, Hiroshima University Hospital, Hiroshima, Japan; 4 Institute for Biomechanics, BG Unfallklinik Murnau, Murnau, Germany; 5 Institute for Biomechanics, Paracelsus Medical Private University Salzburg, Salzburg, Austria; 6 Faculty of Frontier Engineering, Institute of Science & Engineering, Kanazawa University, Kanazawa, Japan; 7 Division of Rehabilitation, Department of Clinical Practice and Support, Hiroshima University Hospital, Hiroshima, Japan; 8 Department of Artificial Joints and Biomechanics, Graduate School of Biomedical and Health Sciences, Hiroshima University, Hiroshima, Japan

**Keywords:** Ultrasound, Three-dimensional motion analysis, Distance between talus and lateral malleolus, Stance phase of gait

## Abstract

**Purpose:**

This study aimed to assess the dynamics of the distance between the talus and lateral malleolus during the stance phase of gait and confirm any sex differences.

**Methods:**

The distance between the talus and lateral malleolus was quantified during gait in 25 healthy participants. Noninvasive ultrasound with three-dimensional motion analysis was used to assess the kinematic properties during the walking stance phase.

**Results:**

The distance between the talus and lateral malleolus widened immediately after initial contact, narrowed during mid-stance, and widened during the stance cycle in all participants. The distance was significantly wider in females, especially during the early and terminal stance phases, contributing to differences in sagittal mobility.

**Conclusions:**

Quantitative measurement of the anterior talofibular ligament attachment distance may aid in the early detection of ankle abnormalities. The dynamic characteristics observed in females during gait may be associated with chronic ankle instability or osteoarthritis risk.

**Supplementary Information:**

The online version contains supplementary material available at 10.1007/s10396-025-01577-6.

## Introduction

Lateral ankle sprains are common sports injuries [1] with a high recurrence rate (18.1–47.0%) and often lead to chronic ankle instability (CAI) [2]. The prevalence of CAI is higher in females than males [3], and the importance of considering sex differences has been highlighted when evaluating mechanical ankle instability.

Evaluating mechanical ankle instability involves assessing laxity in the talocrural and subtalar joints associated with ligamentous damage and kinematic changes in the joint [4]. Ultrasound (US) is widely used to identify anterior talofibular ligament (ATFL) injuries, offering accuracy comparable to that of radiography and magnetic resonance imaging [5]. During US, the ATFL’s origin (lateral malleolus) and insertion (talus) can be drawn on the image to assess the linear dimensions of the ligaments as the distance between the talus and lateral malleolus [6, 7]. Changes in the distance between the talus and lateral malleolus can be evaluated by applying an anterior drawer force and specific stress on the ankle during US examinations [8]. The distance between the talus and lateral malleolus was found to be significantly wider in patients with CAI than in healthy individuals [8], suggesting that repeated ankle sprains can lead to further ligament damage, resulting in joint instability. In healthy individuals, the ATFL ratio, which was calculated as the ratio of stress/nonstress ATFL length during stress US examination, was higher in females than males, potentially showing the high risk of CAI in females [9]. Therefore, evaluating the distance between the talus and lateral malleolus in healthy individuals provides clinically valuable information when considering the risks of CAI.

The main limitation of previous studies was that measurements were performed only under static conditions [8, 10]. As shown in the previous studies, the distance between the talus and lateral malleolus was wider in the ankle plantarflexed and inverted positions [10], and changed with ankle positions. However, mechanical ankle instability has been considered as a problem in dynamic conditions where lateral ankle sprains often occur, showing a gap between the previous studies and real-world clinical problems. Therefore, measuring the distance between the talus and lateral malleolus under dynamic conditions is significant when evaluating the pathophysiology of mechanical ankle instability. We successfully assessed the dynamics of the vertical mobility of the first tarsometatarsal joint during the stance phase of gait using synchronized US imaging and three-dimensional motion analysis (MA) [11, 12].

In the present study, we aimed to use synchronized US imaging and MA to assess the dynamics of the distance between the talus and lateral malleolus during the stance phase of gait and confirm any sex differences. We hypothesized that the distance between the talus and lateral malleolus during the stance phase of gait would be significantly wider in females.

## Material and methods

### Participants

We included 25 healthy adults (13 females, 12 males) who participated in recreational activities, defined as physical exercise at least once a week over the past 2 months without engaging in systematic exercise training. Participants with a Foot Posture Index-6 (FPI-6) score of < 6 were included, while those with a score of ≥ 6 were excluded due to flatfoot alignment [13]. Individuals with a history of ligament injury, plantar fasciitis, synovitis, lower-limb trauma, or neurological conditions affecting the balance of the feet or ankles were excluded. History of ATFL injury, which is one of the most common traumas, was judged based on self-reporting. Prior to gait measurement, the Cumberland Ankle Instability Tool (CAIT) (score range: 0–30) was used to screen all participants for CAI and quantify the perception of ankle joint stability [14]. The maximum CAIT score is 30, with a smaller score indicating more ankle instability. In addition, the Self-Administered Foot Evaluation Questionnaire (SAFE-Q) was used to assess health-related quality of life with a focus on the foot [15]. The SAFE-Q is scored from 0 to 100, with a lower score indicating a poorer foot condition.

This study was approved by the Ethics Committee for Epidemiology of Hiroshima University (Approval Number: E-2187) and was conducted according to the principles of the Helsinki Declaration. Informed consent was obtained from all participants.

### Experimental procedure

#### Assessment of the distance between the talus and lateral malleolus using B-mode US during gait

**Fig. 1:**
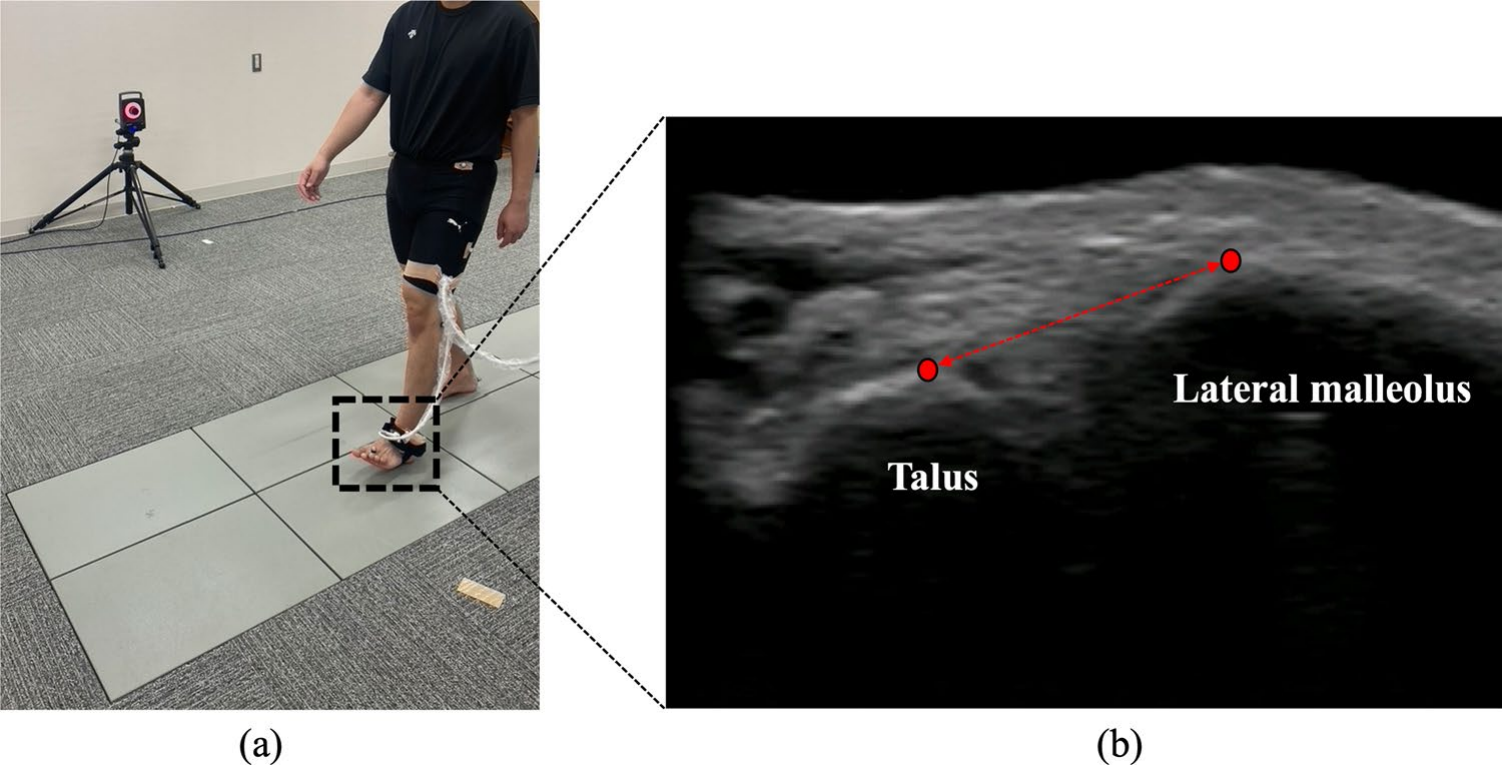
Gait analysis using ultrasound and a three-dimensional motion analysis system. An ultrasound probe was attached to the lateral ankle on an ultrasound gel pad (**a**) to capture the talus and lateral malleolus in the field of view (**b**). The distance between the talus and lateral malleolus was measured as the shortest distance between the talus's lateral neck and the lateral malleolus's anterolateral surface

To assess the distance between the talus and lateral malleolus during the stance phase of gait, we used a B-mode US system (Art Us EXT-1H; Telemed, Vilnius, Lithuania) with a US probe (5–11 MHz, 60 mm field of view; Echoblaster; Telemed, Vilnius, Lithuania). To improve image quality and prevent pressure on the skin, a US gel pad (Yasojima Proceed Co., Ltd., Kobe, Japan) was positioned between the US probe and the skin. The US system was synchronized with the MA system, and B-mode US videos were simultaneously recorded at 80 frames per second, with 60 mm depth, triggered by the start of the former (Fig. 1a). The positioning of the US probe was modified to capture the anterolateral portion of the lateral neck of the talus and lateral malleolus, aligned with the ATFL, in the US image [16]. US videos were visually inspected during each gait test to ensure that the talus and lateral malleolus landmarks were not obscured. Marks were made on each participant's skin to provide a standardized location for the installation of the US probe, and the absence of any displacement was confirmed. US videos were recorded thrice on both sides.

#### Gait analysis

Every participant walked across eight force plates (OR-6, 1000 Hz; AMTI, USA), covering 6 m. Walking patterns were analyzed using a Vicon motion capture system with 16 infrared cameras (100 Hz). Sixteen reflective markers were affixed to the lower body of the participants by the same examiner to ensure consistent placement. The ankle joint angles in the sagittal, transverse, and frontal planes were calculated using the biomechanical Plug-in Gait model of Vicon Nexus ver. 1.8.5 software (Vicon Motion Systems, UK) [17]. The ankle joint angle in the sagittal plane was determined between the foot vector projected onto the sagittal plane of the foot and the shank’s sagittal axis. The angle in the transverse plane was measured for the axis perpendicular to the foot vector and ankle dorsi/plantar flexion axis. In contrast, the angle between the foot vector and the sagittal axis of the shank was projected onto the foot transverse plane. The frontal plane ankle angle was also calculated as the angle between the shank’s foot vector and the frontal axis. After collecting the static standing position data, participants walked across the force plates at their self-selected pace. All participants performed two practice trials before the measurements and took two steps before stepping onto the force plates.

#### Data analysis

The first part of the force plate data was processed using the Plug-in Gait pipeline for the Vicon Nexus ver. 1.8.5 software to perceive the gait phase. The stance phase of gait was defined as the phase from heel contact to toe-off of the foot attached to the US probe. We calculated one stance phase in all three trials on each side. To calculate the distance between the talus and lateral malleolus from the US video, we used Tracker 5.1.5 software (Open Source Physics, https://www.compadre.org). Temporal changes in the distance between the talus and lateral malleolus during the stance phase were calculated using the Vicon software. The distance between the talus and lateral malleolus was determined as the shortest line connecting the tip of each bone (Fig. 1b) and was analyzed in all frames of each stance phase. One stance phase was normalized to 100 frames. The overall profile was analyzed based on the previous activity phases during the gait cycle. Finally, one stance phase between heel contact and toe-off was analyzed separately for three stance phases, as follows: early (0–33 frames), middle (34–66 frames), and terminal (67–100 frames) [18].

### Statistical analysis

The data were analyzed using version 28.0 of the Statistical Package for the Social Sciences (SPSS) for Mac (IBM Corp., Armonk, NY, USA). The normality of the data was confirmed using the Shapiro–Wilk test. A Student’s *t*-test was used to compare quantitative variables. The intra-rater reliability of the distance between the talus and lateral malleolus during the three stance phases was assessed using intraclass correlation coefficients (ICC_1,3_). ICC_1,3_ was considered excellent if > 0.74, suitable if 0.60–0.74, fair if 0.40–0.59, and poor if < 0.40 [19]. Additionally, the measurement standard error (SEM) was calculated for the distance between the talus and lateral malleolus at each stance phase for both sexes to assess measurement accuracy.

Two-way split-plot analysis of variance (ANOVA) was used to determine differences in US values and average ankle angles in each of the three dimensions between sexes as a between-participant factor and stance phase as a within-participant factor. Post hoc comparisons were conducted using the Tukey method when interaction effects were detected. Partial η2 values were used to measure the effect size. The post hoc observed power based on partial η2 was generated using G*Power 3.1 software (Kiel University, Germany). A sample size of 26 feet in the female group and 24 feet in the male group was used for the statistical power analysis. The post hoc analysis results showed a significant effect size (*d* = 0.8), an alpha level of *p* < 0.05, and a statistical power of 0.873, indicating adequate power. Statistical significance was set at *p* < 0.05.

## Results

The demographic characteristics of participants are presented in Table 1. The mean height (*p* < 0.001) and body weight (*p* = 0.002) were significantly lower in females. No significant differences in CAIT, FPI-6, and SAFE-Q scores were found between sexes (Table 1). The intra-rater reliability for the US distance between the talus and lateral malleolus during each stance phase was considered excellent (Table 2).

**Table 1:** General characteristics of healthy participants

	Females (*n* = 13, 26 feet)	Males (*n* = 12, 24 feet)	*p*-value
Age (years)	21.3 ± 1.4	22.1 ± 1.0	0.166
Height (cm)	158.4 ± 4.4	170.4 ± 6.2	< 0.001
Body weight (kg)	51.6 ± 5.7	63.0 ± 9.8	0.002
Body mass index (kg/m^2^)	20.1 ± 1.8	22.9 ± 3.4	0.235
Cumberland Ankle Instability Tool	28.8 ± 1.6	27.8 ± 3.6	0.688
Foot posture index	3.1 ± 1.2	2.9 ± 0.9	0.306
Self-Administered Foot Evaluation Questionnaire			
Pain and pain-related	94.5 ± 13.1	97.3 ± 5.8	0.026
Physical functioning and daily living	97.6 ± 6.0	99.6 ± 1.3	0.129
Social functioning	98.7 ± 3.6	99.3 ± 2.4	0.318
Shoe-related	95.5 ± 10.0	94.4 ± 14.4	0.415
General health and well-being	98.1 ± 5.6	98.3 ± 5.8	0.456

Data are shown as mean ± standard deviation

**Table 2:** Reproducibility of the ultrasound movement of the distance between the talus and lateral malleolus during the stance phase of gait

		Females		Males	
	Stance phase	ICC _1,3_	SEM	ICC _1,3_	SEM
The distance between the talus and lateral malleolus	Early phase	0.909 (0.743–0.957)	0.216	0.949 (0.854–0.986)	0.349
	Middle phase	0.899 (0.712–0.972)	0.310	0.929 (0.799–0.981)	0.255
	Terminal phase	0.895 (0.702–0.971)	0.314	0.931 (0.806–0.981)	0.280

*ICC* intraclass correlation coefficient, *SEM* standard error of the measurements

SEM was calculated using the formula s√1-ICC. Values in parentheses are 95% confidence for ICC and lower and upper limits for SEM

Table 3 shows the results of a two-way split-plot ANOVA, with sex as the between-subject factor and t the distance between the talus and lateral malleolus and ankle angles during the three phases of gait. A significant interaction effect was observed for the distance between the talus and lateral malleolus (*F* = 3.698, *p* = 0.027, partial *η*^2^ [$\eta_{{\text{p}}}^{2}$] = 0.049). A main effect of sex was observed for transverse plane ankle and frontal plane ankle angles. Additionally, a main effect of the stance phase was observed for sagittal plane ankle angles.

**Table 3:** Differences in the distance between the talus and lateral malleolus, sagittal plane ankle angles, transverse plane ankle angles, and frontal plane ankle angles during the stance phase

	Interaction (sex × phase)				Main effect (sex)				Main effect (phase)			
	*F*	*p*	*η*^2^	Observed power	*F*	*p*	*η*^2^	Observed power	*F*	*p*	*η*^2^	Observed power
Distance between talus and lateral malleolus	3.698	0.027	0.049	0.671	18.866	< 0.001	0.116	0.991	31.187	< 0.001	0.302	1.000
Sagittal plane ankle angles	0.756	0.471	0.010	0.177	2.639	0.106	0.018	64.119	64.119	< 0.001	0.471	1.000
Transverse plane ankle angles	0.024	0.976	0.000	0.054	51.562	< 0.001	0.264	1.000	4.341	0.015	0.057	0.745
Frontal plane ankle angles	0.017	0.983	0.000	0.053	37.196	< 0.001	0.205	1.000	3.927	0.022	0.052	0.699

Data are shown as mean ± standard deviation

*d* Cohen’s *d*, *η*^2^ partial eta-squared

A significant effect of participant sex on the distance between the talus and lateral malleolus was found. Significant effects of the gait phase on the distance between the talus and lateral malleolus, sagittal plane ankle angles, transverse plane ankle angles, and frontal plane ankle angles were found

**Fig. 2:**
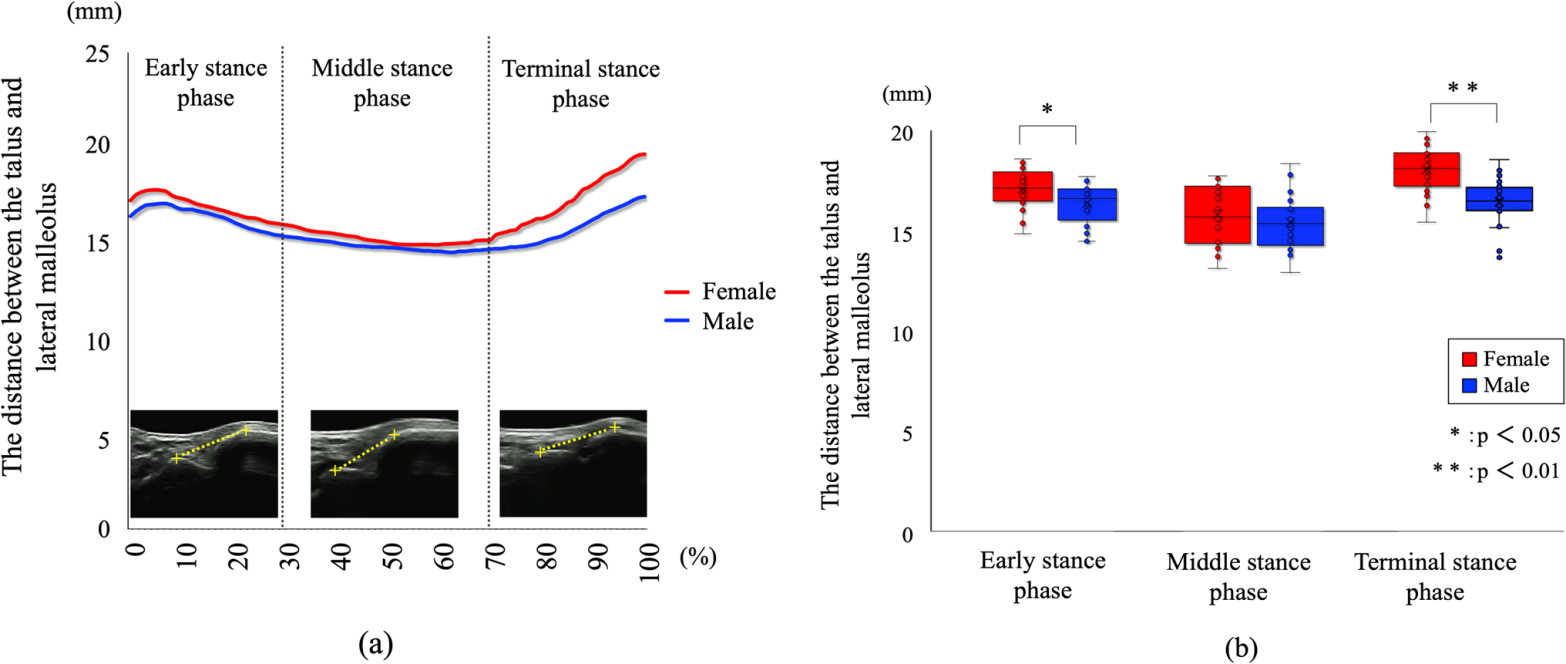
Sex differences in the distance between the talus and lateral malleolus during one stance phase of gait. This shows the calcaneus-fibula lateral malleolus distance transition within one gait cycle (**a**). Box plots are shown to compare vertical locations during the early, middle, and terminal stance phases (**b**). *p < 0.05, **p < 0.01. Compared to male participants, females exhibited a significant increase in the distance between the talus and lateral malleolus during the early and terminal stance phases

The changes in the ankle joint angles of dorsiflexion–plantar flexion during the stance phase of gait between the groups with and without the US probe were not significantly different. The distance between the talus and lateral malleolus widened immediately after the initial contact but gradually narrowed. In the terminal stance phase, the distance increased gradually. Additionally, the distance between the talus and lateral malleolus was more significant in females during the early and terminal stance phases (Fig. 2; *p* < 0.05 and *p* < 0.01, respectively). In the early phases, the distance between the talus and lateral malleolus in females was 16.90 ± 1.09 mm, and it was 3.3% wider than that of males. Also, in the terminal stance phases, the distance between the talus and lateral malleolus in females was 17.36 ± 1.11 mm, and there was a 9.9% difference between females and males. The distance between the talus and lateral malleolus remained stable during the mid-stance phase, with no significant difference between sexes (Fig. 2, *p* = 0.29).

**Fig. 3:**
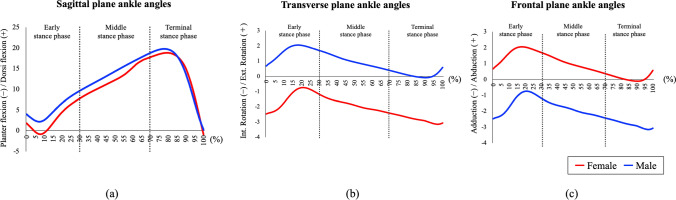
Graphs of ankle angles during one gait cycle. Sagittal plane ankle angles (**a**), transverse plane ankle angles (**b**), and frontal plane ankle angles (**c**)

Sex significantly affected the distance between the talus and lateral malleolus, as well as the transverse and frontal plane ankle angles, during the stance phase (all *p* < 0.001). Figure 3 shows changes in ankle angles during the stance phase of gait. The three stance phases had a significant effect on the distances between the sagittal, transverse, and frontal plane ankle angles during the stance phase (*p* < 0.001, *p* < 0.001, *p* < 0.05, and *p* < 0.05, respectively; Table 3).

## Discussion

This study demonstrated that the distance between the talus and lateral malleolus widened immediately after the initial contact, narrowed during the middle stance phase, and widened again during the terminal stance phase in both groups. Furthermore, the distance increased from the early-to-middle stance and decreased from the middle-to-terminal stance. It has been reported that the distance between the talus and lateral malleolus decreases as the ankle dorsiflexes, the talus moves posteriorly, and the distance increases as the ankle plantar flexes [20]. The change in the distance between the talus and lateral malleolus during stance phase in gait is considered to be a response to the movement of the talus with the ankle joint movement in the dorsi/planter flexion direction, and observations in the present study are based on anatomical reports [20, 21]. This may reflect dynamic behavior similar to what has been verified under normal nonweight-bearing conditions.

During the middle stance phase, the distance did not differ between sexes; however, the distance was wider in females during the early and terminal stance phases. Compared with males, the ankle joint in healthy females has increased talar dorsiflexion, internal rotation, and internal rotation angles of the subtalar joint in the early stance phase of gait [21]. Adding to well-known knowledge of sex-based kinematic differences in gross motion while walking, the report showed that the sex-based differences exist specifically in talocrural and subtalar joint kinematics during walking [21]. Furthermore, the result in the present study can be considered to be related to the previous reports, and provides new findings on the sex-based kinematic differences from the anatomical perspective in the distance changes between the talus and lateral malleolus.

A previous study using dynamic biplane fluoroscopy showed that females exhibited greater anteroposterior mobility of the tibiotalar joint compared to males [22]. This finding is considered to be associated with anatomical characteristics specific to females. It has been documented that females, compared to males, have greater ligamentous laxity of the lateral ankle ligaments and the deltoid ligament, resulting in reduced passive support of the ankle joint [23, 24]. Furthermore, Tashiro et al. reported that the distal tibiofibular joint showed significantly greater widening under weight-bearing conditions in females than in males [25], and that this widening may be associated with instability of the tibiotalar joint [26]. These anatomical sex differences in the distal tibiofibular joint may contribute to decreased rigidity of the tibiotalar joint, potentially leading to an increased distance between the talus and lateral malleolus during gait. The present findings are consistent with these previous reports and suggest that sex-specific differences in joint structures and supporting mechanisms may influence dynamic ankle kinematics during gait. Furthermore, previous studies employing dynamic measurements have often utilized dynamic biplane fluoroscopy [27]; however, limitations such as radiation exposure and restrictions on foot placement have been reported. In contrast, the present study offers a novel insight by enabling measurements in a manner closer to natural gait through the synchronization of US and MA systems.

This study had several limitations. First, the participants were limited to young, healthy adults. Since kinematic characteristics during gait may vary depending on age or sports history, it is necessary to further clarify ankle joint motion in more diverse populations. Second, although dynamic sex differences in the distance between the talus and lateral malleolus were identified, anatomical factors such as the ATFL, which may influence these dynamics, were not examined. The ATFL plays an important role in controlling the anterior movement of the talus, and future studies should investigate such anatomical elements. Lastly, the potential influence of the US probe attachment on ankle joint kinematics during gait should be taken into consideration.

In this study, a novel method was applied that synchronized US with an MA system to evaluate changes in the distance between the talus and lateral malleolus and ankle joint kinematics in a dynamic, weight-bearing gait environment. This approach enabled detailed assessment of gait phase-dependent changes and successfully revealed sex-based differences in ankle joint motion.

## Conclusion

In conclusion, partial mobility of the ankle joint during the stance phase of gait was quantified by synchronizing US and MA. Compared with males, in healthy females without foot impairment, the distance between the talus and lateral malleolus was more significant during the early and terminal phases of the stance. These findings suggest that this approach may be applicable in future studies for advancing our understanding of the pathophysiology of foot and ankle disorders, such as chronic ankle instability and osteoarthritis, through comparative analyses.

## Supplementary Information

Below is the link to the electronic supplementary material.

Supplementary file1 (DOCX 115 KB)

## Data Availability

The datasets for the present study are available from the corresponding author upon request.

## References

[1] Roos KG, Kerr ZY, Mauntel TC, et al. The epidemiology of lateral ligament complex ankle sprains in National Collegiate Athletic Association sports. Am J Sports Med. 2017;45:201–9. 27573356 10.1177/0363546516660980

[2] Herzog MM, Kerr ZY, Marshall SW, et al. Epidemiology of ankle sprains and chronic ankle instability. J Athl Train. 2019;54:603–10. 31135209 10.4085/1062-6050-447-17 PMC6602402

[3] Mendes R, Silva A, Santos P. The impact of physical activity on lower limb strength and balance in older adults: a systematic review. J Foot Ankle Res. 2022;15:41.35624522

[4] Hubbard TJ, Hertel J. Mechanical contributions to chronic lateral ankle instability. Sports Med. 2006;36:263–77. 16526836 10.2165/00007256-200636030-00006

[5] Cao S, Wang C, Ma X, et al. Imaging diagnosis for chronic lateral ankle ligament injury: a systemic review with meta-analysis. J Orthop Surg Res. 2018;13:122. 29788978 10.1186/s13018-018-0811-4 PMC5964890

[6] Abdeen R, Comfort P, Starbuck C, et al. Ultrasound characteristics of foot and ankle structures in healthy, coper, and chronically unstable ankles. J Ultrasound Med. 2019;38:917–26. 30208221 10.1002/jum.14770

[7] Kikumoto T, Akatsuka K, Nakamura E, et al. Quantitative evaluation method for clarifying ankle plantar flexion angles using anterior drawer and inversion stress tests: a cross-sectional study. J Foot Ankle Res. 2019;12:27. 31073333 10.1186/s13047-019-0337-y PMC6500013

[8] Kristen KH, Seilern und Aspang J, Wiedemann J, et al. Reliability of ultrasonography measurement of the anterior talofibular ligament (ATFL) length in healthy subjects (in vivo), based on examiner experience and patient positioning. J Expo Orthop. 2019;2:30. 10.1186/s40634-019-0199-z PMC6606687 31267337

[9] Yokoe T, Tajima T, Kawagoe S, et al. The ratio of stress to nonstress anterior talofibular ligament length on ultrasonography: normative values. Orthop J Sports Med. 2021;9:23259671211056305. 34820463 10.1177/23259671211056305 PMC8607488

[10] Croy T, Saliba SA, Saliba E, et al. Differences in lateral ankle laxity measured via stress ultrasonography in individuals with chronic ankle instability, ankle sprain copers, and healthy individuals. J Orthop Sports Phys Ther. 2012;42:593–600. 22446334 10.2519/jospt.2012.3923

[11] Maeda N, Ikuta Y, Tasiro T, et al. Quantitative evaluation of the vertical mobility of the first tarsometatarsal joint during the stance phase of gait. Sci Rep. 2022;12:9246. 35655091 10.1038/s41598-022-13425-5 PMC9163033

[12] Tashiro T, Ikuta Y, Maeda N, et al. First tarsometatarsal joint mobility in hallux valgus during gait: a synchronized ultrasound and three-dimensional motion capture analysis. J Med Ultrason. 2024;51:331–9. 10.1007/s10396-024-01414-2 PMC11098882 38546904

[13] Gijon-Nogueron G, Martinez-Nova A, Alfageme-Garcia P, et al. International normative data for paediatric foot posture assessment: a cross-sectional investigation. BMJ Open. 2019;9:e023341. 30987983 10.1136/bmjopen-2018-023341 PMC6500282

[14] Wright CJ, Arnold BL, Ross SE, et al. Recalibration and validation of the Cumberland Ankle Instability Tool cutoff score for individuals with chronic ankle instability. Arch Phys Med Rehabil. 2014;95:1853–9. 24814563 10.1016/j.apmr.2014.04.017

[15] Niki H, Haraguchi N, Aoki T, et al. Responsiveness of the self-administered foot evaluation questionnaire (SAFE-Q) in patients with hallux valgus. J Orthop Sci. 2017;22:737–42. 28501433 10.1016/j.jos.2017.04.005

[16] Özgül B, Starbuck C, Polat MG, et al. Inter and intra-examiner reliability of musculoskeletal ultrasound scanning of anterior talofibular ligament and ankle muscles. J Ultrasound. 2023;26:137–46. 36048331 10.1007/s40477-022-00699-8 PMC10063751

[17] Vicon Motion Systems Ltd. Plug-in Gait Reference Guide. https://help.vicon.com/space/Nexus215/11379858/Plug-in+Gait+kinematic+variables. Accessed 2023.

[18] Kim EJ, Shin HS, Takatori N, et al. Inter-segmental foot kinematics during gait in elderly females according to the severity of hallux valgus. J Orthop Res. 2020;38:2409–18. 32162717 10.1002/jor.24657

[19] Landis JR, Koch GG. The measurement of observer agreement for categorical data. Biometrics. 1977;33:159–74. 843571

[20] Khawaji B, Soames R. The anterior talofibular ligament: a detailed morphological study. Foot. 2015;25:141–7. 26205996 10.1016/j.foot.2015.05.004

[21] Fukano M, Fukubayashi T, Banks SA. Sex differences in three-dimensional talocrural and subtalar joint kinematics during stance phase in healthy young adults. Hum Mov Sci. 2018;61:117–25. 30086450 10.1016/j.humov.2018.06.003

[22] Yang S, Canton SP, Hogan MV, et al. Healthy ankle and hindfoot kinematics during gait: sex differences, asymmetry, and coupled motion revealed through dynamic biplane radiography. J Biomech. 2021;116:110220. 33422727 10.1016/j.jbiomech.2020.110220 PMC7878402

[23] Wilkerson RD, Mason MA. Differences in men’s and women’s mean ankle ligamentous laxity. Iowa Orthop J. 2000;20:46–8. 10934624 PMC1888743

[24] Kato E, Oda T, Chino K, et al. Musculotendinous factors influencing difference in ankle joint flexibility between women and men. Int J Sport Health Sci. 2005;3:218–25.

[25] Tashiro T, Maeda N, Tsutsumi S, et al. A quantitative assessment of the anterior tibiofibular gap with and without weight-bearing in healthy adults: an ultrasound-based study. J Orthop Sci. 2025;30:107–12. 38388331 10.1016/j.jos.2024.01.008

[26] Vohra R, Singh A, Thorat B, et al. Instability of the distal tibiofibular syndesmosis. J Orthop Surg. 2023;31:10225536231182349. 10.1177/10225536231182349 37449812

[27] Cao S, Wang C, Zhang G, et al. In vivo kinematics of functional ankle instability patients during the stance phase of walking. Gait Posture. 2019;73:262–8. 31382233 10.1016/j.gaitpost.2019.07.377

