# Supplementary material for: Sex differences in the dynamics of the distance between the talus and lateral malleolus during the stance phase of gait
Source: J Med Ultrason (2001). 2025 Sep 18;53(1):19–26. doi: 10.1007/s10396-025-01577-6 (PMC12790505; doi:10.1007/s10396-025-01577-6)
Supplement: Supplementary file 1 — Supplementary file1 (DOCX 115 KB) [file 10396_2025_1577_MOESM1_ESM.docx]

**Supplementary data**

To investigate the possibility that the attachment of the probe could ankle motion, the dorsiflexion and plantarflexion angles of the ankle during the stance phase of gait were compared with and without the probe. A paired t-test was used to compare the ankle angles during the stance phase with and without the probe. The results showed no significant differences between the two conditions (Supplementary Fig. 1).


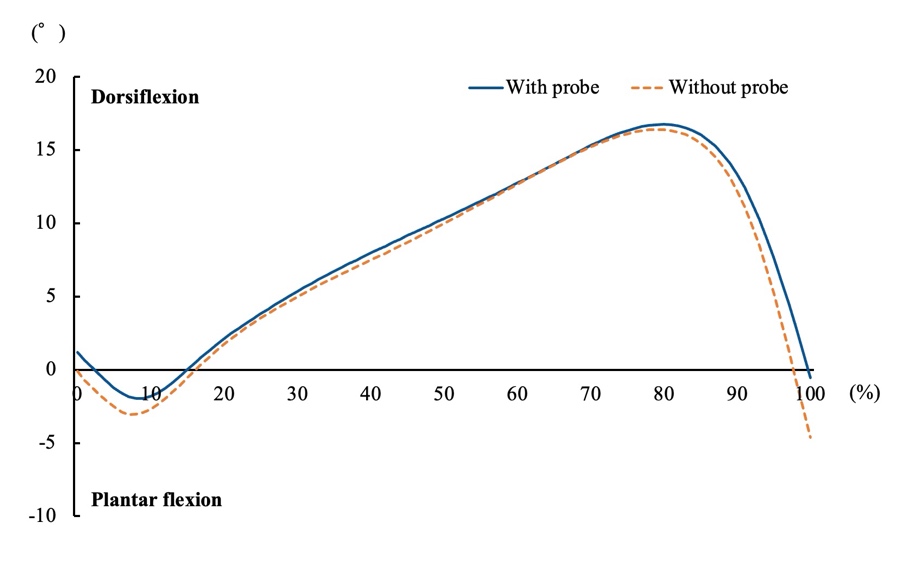


(a)


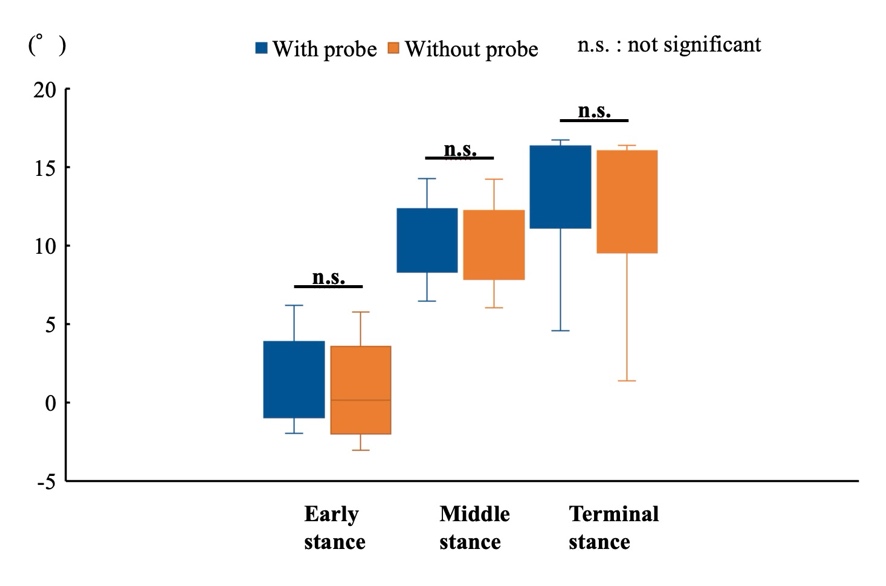


(b)

Supplemental Figure 1. The ankle dorsiflexion/ plantarflexion angles during one stance phase of gait (a). Box plots compare ankle angles during the early, middle, and terminal stance phases (b). n.s.: not significant.
